# Supplementary material for: Dual Labeling of Primary Cells with Fluorescent Gadolinium Oxide Nanoparticles
Source: Nanomaterials (Basel). 2023 Jun 16;13(12):1869. doi: 10.3390/nano13121869 (PMC10304197; doi:10.3390/nano13121869)
Supplement: Supplementary file 1 [file nanomaterials-13-01869-s001.zip › nanomaterials-2388312-supplementary.pdf]

Supporting Information

Dual labeling of primary cells with fluorescent gadolinium oxide nanoparticles

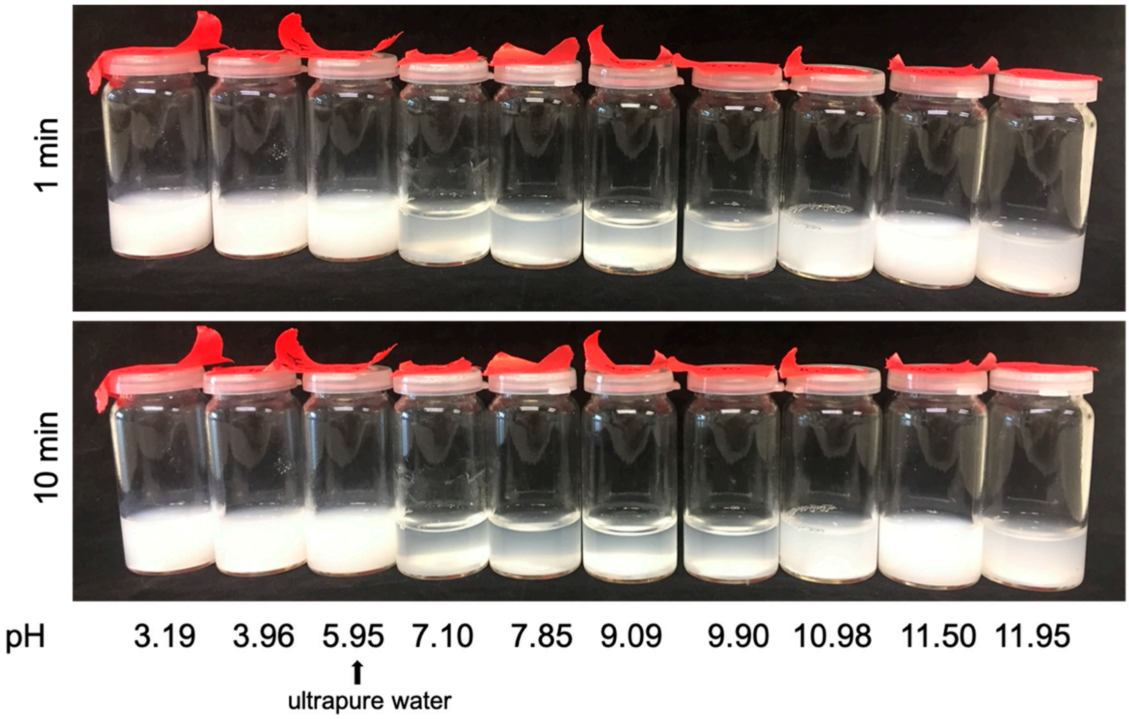

Figure S1. Gd_2O_3 nanoparticles dispersed in pH series. The ultrapure water has a pH of 5.95. Representative images are shown for 1 min and 10 min after dispersion.

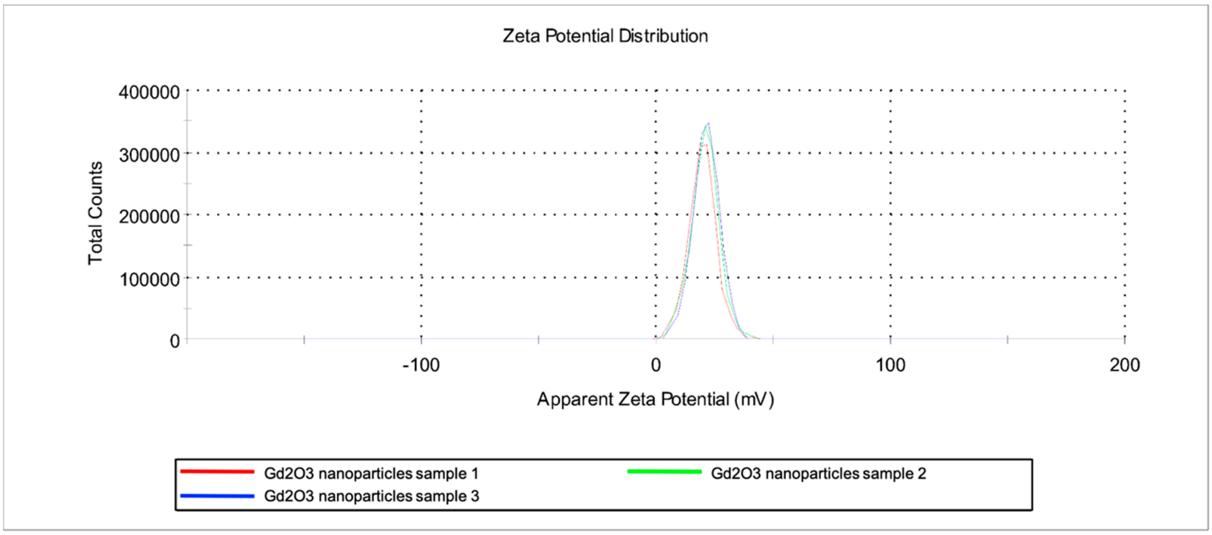

Figure S2. Apparent Zeta-potential in mV (x-axis) for triplicate samples of 10 mM Gd_2O_3 nanoparticles in UPW consisting of averaged data from 12 runs each. Mean Zeta Potential = 19.8 ± 5.9 mV.

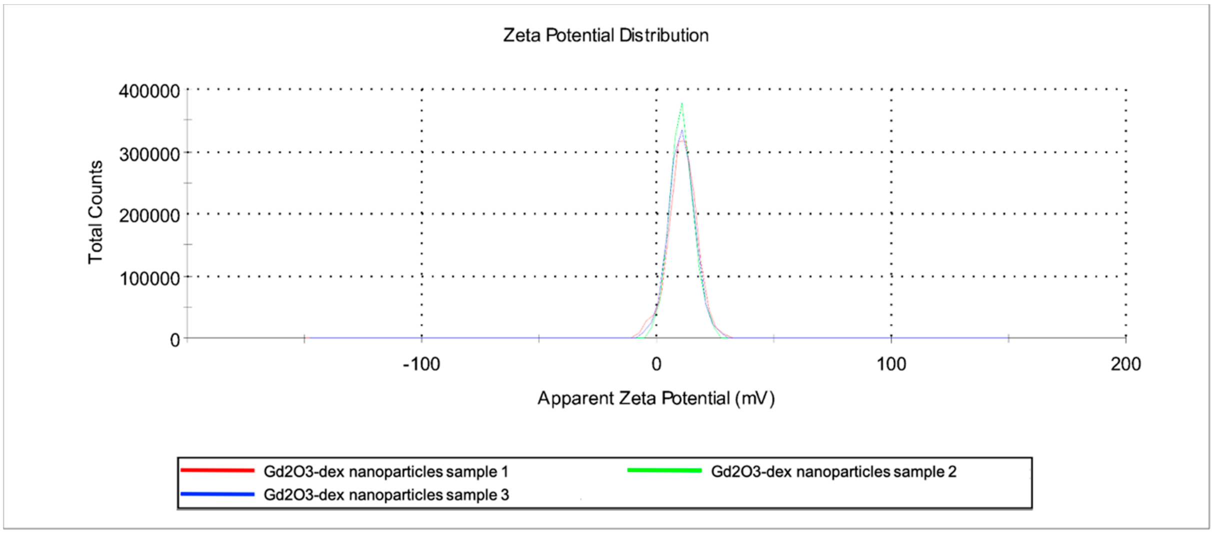

Figure S3. Apparent Zeta-potential in mV (x-axis) for triplicate samples of 10 mM Gd_2O_3-dex nanoparticles in UPW consisting of averaged data from 12 runs each. Mean Zeta Potential = 11.1 ± 6.0 mV.

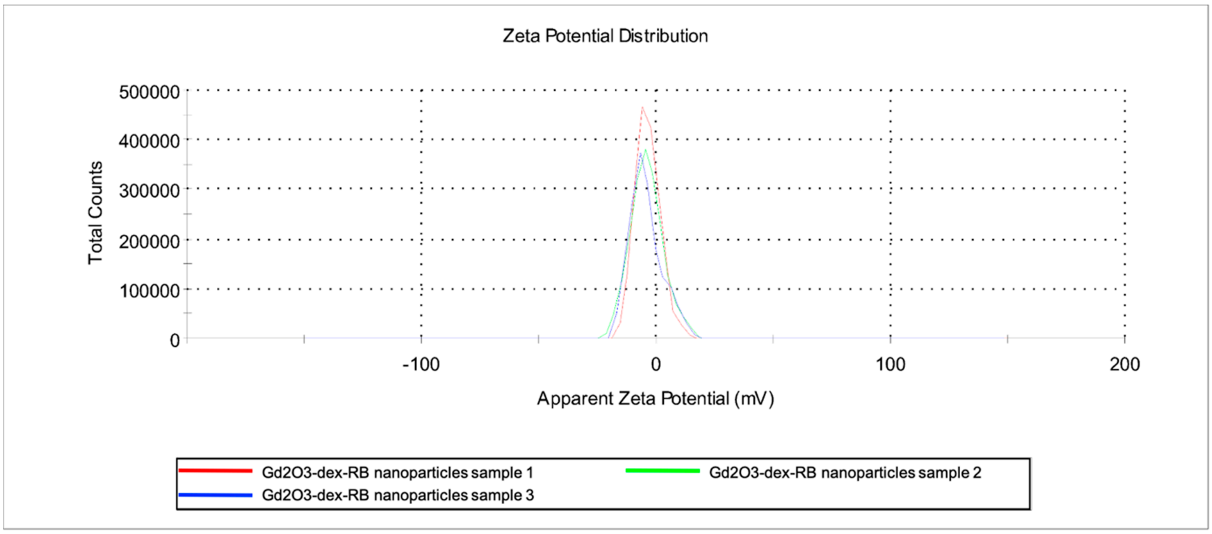

Figure S4. Apparent Zeta-potential in mV (x-axis) for triplicate samples of 10 mM Gd_2O_3-dex-RB nanoparticles in UPW consisting of averaged data from 12 runs each. Mean Zeta Potential = -3.52 ± 5.45 mV.

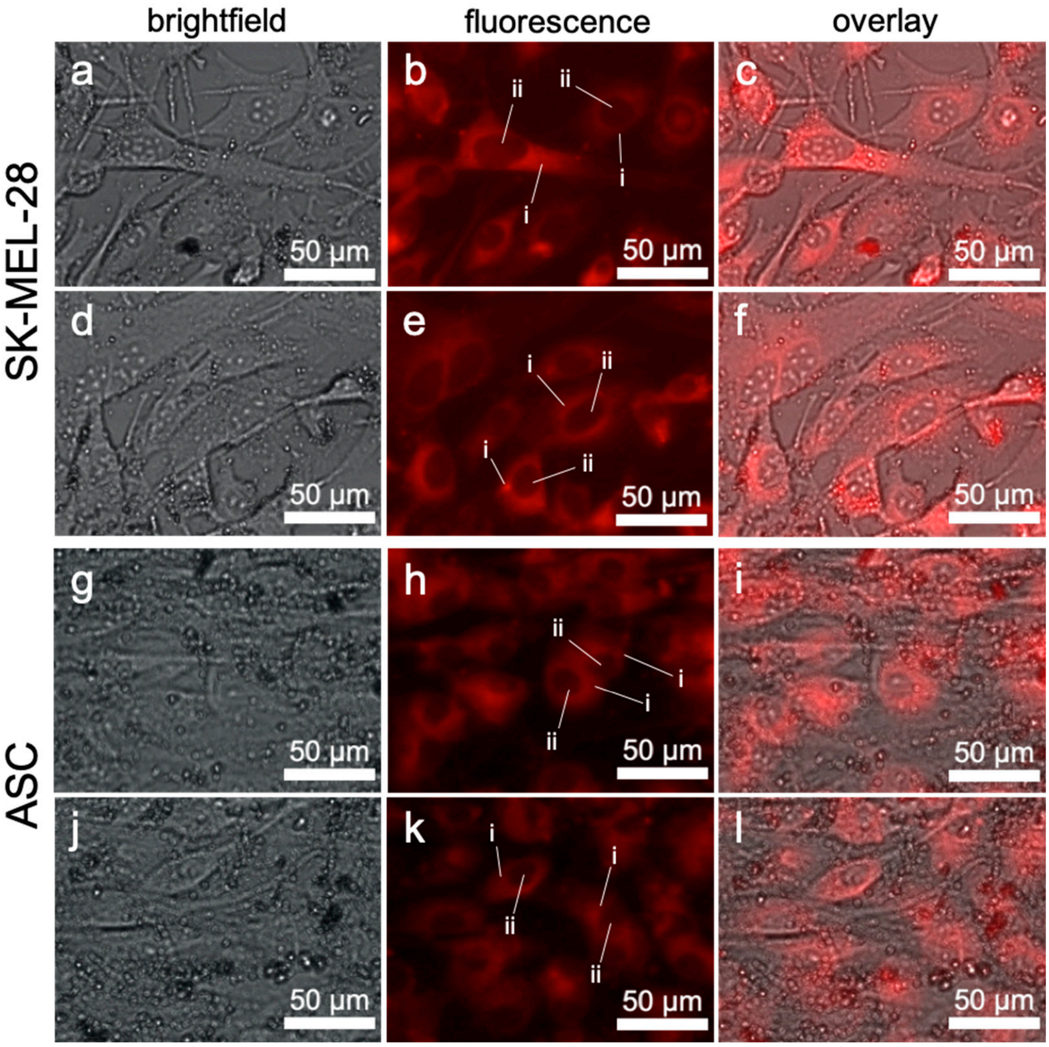

Figure S5. SK-MEL-28 (**a**-**f**) and ASC (**g**-**l**) incubated with 2 mM Gd_2O_3-dex-RB nanoparticles for 24 h, respectively. Showing bright-field (**a**, **d**, **g**, **j**), fluorescence microscopy (**b**, **e**, **h**, **k**), and overlay (**c**, **f**, **i**, **l**). Both cell lines show a bright fluorescence signal in the cytosol (i), sparing the nucleus (ii).
